# A recombinant *Artemisia vulgaris* pollen adjuvanted Art v 1 protein-based vaccine treats allergic rhinitis and bronchial asthma using pre- and co-seasonal ultrashort immunotherapy regimens in sensitized mice

**DOI:** 10.3389/fimmu.2022.983621

**Published:** 2022-11-09

**Authors:** Meruert Babayeva, Kairat Tabynov, Tair Nurpeisov, Gleb Fomin, Gourapura J. Renukaradhya, Nikolai Petrovsky, Kaissar Tabynov

**Affiliations:** ^1^ International Center for Vaccinology, Kazakh National Agrarian Research University (KazNARU), Almaty, Kazakhstan; ^2^ Department of General Immunology, Asfendiyarov Kazakh National Medical University (KazNMU), Almaty, Kazakhstan; ^3^ Preclinical Research Laboratory with Vivarium, M. Aikimbayev National Research Center for Especially Dangerous Infections, Almaty, Kazakhstan; ^4^ T&TvaX LLC, Almaty, Kazakhstan; ^5^ Center for Food Animal Health, Ohio Agricultural Research and Development Center, The Ohio State University (OSU), Wooster, OH, United States; ^6^ Vaxine Pty Ltd, Flinders University, Bedford Park, SA, Australia; ^7^ Republican Allergy Center, Research Institute of Cardiology and Internal Medicine, Almaty, Kazakhstan

**Keywords:** allergy, vaccine, allergen-specific immunotherapy, wormwood pollen, Art v 1 protein, adjuvant, mice

## Abstract

Allergic rhinitis is an important risk factor for bronchial asthma. Allergen-specific immunotherapy (ASIT) is the gold standard for treatment of allergic rhinitis, conjunctivitis, and asthma. A disadvantage of current ASIT methods is the length of therapy which requires numerous allergen administrations. The success of ASIT is determined by its schedule, which, depending on the vaccine and type of allergy, can be pre-seasonal (before the allergy season begins), combined pre/co-seasonal (during the allergy season) etc. The aim of the present study was to evaluate a vaccine based on recombinant *Artemisia vulgaris* pollen major Art v 1 protein formulated with ISA-51 adjuvant for therapy of allergic rhinitis and bronchial asthma in *Artemisia*-sensitized mice in an ultrashort (4 subcutaneous injections at weekly intervals) pre- and co-seasonal ASIT regimen.

To simulate co-seasonal ASIT in mice, mice were regularly challenged with intranasal and nebulized *Artemisia vulgaris* pollen extract at the same time as receiving subcutaneous ASIT. For comparison, we used a previous Art v 1 protein vaccine formulated with SWE adjuvant, which in this study was modified by adding CpG oligonucleotide (Th1-biasing synthetic toll-like receptor 9 agonist), and a commercial vaccine containing a modified *Artemisia vulgaris* extract with aluminum hydroxide adjuvant. The therapeutic potential of Art v 1 based vaccine formulations with different ASIT regimens was evaluated in high and low (10 times lower) dose regimens.

The ISA-51-adjuvanted vaccine formulations were the only ones among those studied in the ultrashort pre- and co-seasonal ASIT regimens to provide significant reduction in both signs of allergic rhinitis and bronchial asthma in sensitized mice (vs. positive control). In the ISA-51 adjuvanted group, immune response polarization toward Th1/Treg was observed in pre-seasonal ASIT, as reflected in a significant decrease in the serum level of total and Art v 1-specific IgE and increased ratios of allergen-specific IgG2a/IgG1 and IFN-γ/IL-4. The high dose SWE-CpG-adjuvanted vaccine had similar efficacy to the ISA-51 adjuvanted groups whereas the commercial vaccine showed significantly less effectiveness.

The findings support further preclinical safety studies of the Art v 1-based vaccine formulated with ISA-51 adjuvant.

## Introduction

The prevalence of IgE-mediated allergic diseases in industrialized countries has reached 35% (1), with experts predicting a further increase in coming decades (2, 3). Allergen-specific immunotherapy (ASIT) was first performed by Noon in 1911 (4), and to this day is the gold standard in the therapy of patients with allergic rhinitis, conjunctivitis, and asthma (type I allergies) (2, 5). ASIT, in contrast to symptomatic methods of allergy treatment, induces a restructuring of undesirable allergen-specific humoral and T-cell responses from Th2 to mixed Th1/Treg and thus has a therapeutic disease-modifying effect (6). The essence of ASIT is to gradually introduce increasing amounts of an allergen to a patient with IgE-mediated allergic disease in order to alleviate the symptoms arising from subsequent contact with the causative allergen (5).

ASIT has traditionally been performed by subcutaneous administration of soluble allergen extracts or their adjuvanted aluminum hydroxide or aluminum phosphate forms (5, 7). Subcutaneous ASIT after a full course provides a long-term therapeutic effect against many types of allergens (2, 5). Subcutaneous is more effective than sublingual, ASIT (8), although sublingual therapy has increased in popularity due to its easier administration and better safety profile (9). However, a common significant disadvantage of subcutaneous and sublingual methods of ASIT is that the course of therapy is excessively long (3 to 5 years) requiring very large numbers of administrations. This creates issues of compliance (patients not completing the full course of therapy), but also increases the chance of adverse events (10).

Our previous studies in mice (11) showed that it is possible to achieve effective therapy of bronchial asthma caused by wormwood pollen (*Artemisia*), one of the ten global aeroallergens (12), with only four injections (at weekly intervals) of a vaccine based on the recombinant Art v 1 *Artemisia vulgaris* major protein formulated with ISA-51 adjuvant. However, it is not known whether this vaccine formulation in an ultrashort ASIT regimen can be effective in the treatment of allergic rhinitis, the presence of which has been recognized as an important risk factor for bronchial asthma (13). Patients with allergic rhinitis are three times more likely to develop asthma and relief of rhinitis symptoms over time correlates with improvement of asthma symptoms (14, 15).

The success of ASIT is largely determined by its schedule, which, depending on the vaccine and type of allergy, can be continuous year-round, pre-seasonal (before the allergy season begins), or combined pre/co-seasonal (during the allergy season) (16). The ultrashort ASIT regimen is most suitable for the treatment of pollen allergies before the allergy season begins (17). Nevertheless, there are often situations in which it is not possible to perform ASIT before the allergy season, in which case it is crucial to be able to perform therapy during the allergy season as well. Considering that in many countries (18, 19), including Kazakhstan (20), the wormwood flowering period (from July to October) is long enough, the possibility of conducting co-seasonal ASIT is of great practical importance.

Based on the above, the present studies aimed to evaluate the previously developed vaccine based on the *Artemisia vulgaris* major recombinant protein Art v 1 with ISA-51 adjuvant for therapy of allergic rhinitis and bronchial asthma in *Artemisia-*sensitized mice using ultrashort pre- and co-seasonal ASIT regimens. For modeling co-seasonal ASIT in mice, challenge by intranasal administration and nebulization of wormwood pollen extract was performed alongside desensitizing vaccine immunization. For comparison, we used a previously studied but less effective SWE adjuvanted vaccine (11), which in this experiment was improved by adding CpG (Th1-biasing synthetic toll-like receptor 9 agonist), and a commercial vaccine containing a modified *Artemisia vulgaris* extract with aluminum hydroxide adjuvant. The therapeutic potential of Arv v 1 based vaccine formulations in different ASIT regimens was evaluated in high and low (10 times lower) dose regimens.

## Materials and methods

### Vaccine formulations preparation

The studies used a commercial recombinant major wormwood pollen protein Art v 1 (AtaGenix laboratories, China; Expressed Host - *E. coli*, Purity - 90% as determined by SDS-PAGE quantitative densitometry by Coomassie Blue Staining; Art v 1 - 129AAs, 22 kDa, protein concentration - 2 mg/vial, Endotoxin - 86 EU/mg) plus as adjuvants Montanide ISA-51 VG (ISA-51; water-in-oil emulsion type, Seppic, France) and Sepivac SWE™ adjuvant mixture (SWE; oil-in-water emulsion type, Seppic) with CpG55.2 oligonucleotide (Vaxine Pty Ltd, Australia), from which four vaccine formulations were prepared (**Table 1**). Two-fold increasing concentrations of Art v 1 protein were used for high (from 2 to 16 µg/dose) and low (from 0.2 to 1.6 µg/dose) dose regimens of ASIT, which were formulated with adjuvants in a 50:50 ratio (by volume). Emulsification of the antigen with ISA-51 was performed according to the recommendation of the adjuvant manufacturer using IKA^®^ ULTRA-TURRAX^®^ disperser tubes with rotor-stator element DT-50-M-gamma (IKA^®^-Werke GmbH & Co. KG, Germany). The process of emulsification of the vaccine with ISA-51 was performed in a volume of 50 mL at 20-22°C in two phases: pre-emulsification at low shear rate (1100 rpm for 2 min) and emulsification at high shear rate (4000 rpm for 10 min). The mixture of antigen and SWE-CpG adjuvant was stirred using a laboratory vortex for 10 s. The prepared vaccine formulations were poured into vials and stored at 2-8°C until use. All vaccine formulations were kept sterile and contained less than 2 EU endotoxin content per high allergen dose regimen.

**Table 1:** Vaccine formulations for allergen-specific immunotherapy.

Formulation	Antigen concentration per 100 µL (dose) of vaccine, µg*	Concentration/volume of adjuvant per 100 µL (dose) of vaccine	Method of administration
**ISA-51 High dose**	2, 4, 8, 16	50 µL	s/c
**ISA-51 Low dose**	0.2, 0.4, 0.8, 1.6	50 µL	s/c
**SWE-CpG High dose**	2, 4, 8, 16	50 µL (SWE) + 10 µg (CpG)	i/m
**SWE-CpG Low dose**	0.2, 0.4, 0.8, 1.6	50 µL (SWE) + 10 µg (CpG)	i/m

* Antigen concentrations for 1^st^, 2^nd^, 3^rd^, and 4^th^ immunizations, respectively; s/c – subcutaneous; i/m – intramuscular.

A commercial CLUSTOID^®^ vaccine based on a treated *Artemisia vulgaris* extract formulated with aluminum hydroxide adjuvant (10,000 Therapeutic units [TU]/mL, ROXALL Medizin GmbH, Hamburg, Germany) was used as a comparison drug. For 1^st^, 2^nd^, 3^rd^, and 4^th^ immunizations the commercial vaccine was administered subcutaneously at doses of 200 TU/dose (or 20 µl), 400 TU/dose (40 µl), 800 TU/dose (80 µl), and 1600 TU/dose (160 µl), respectively. PBS was administered in a similar manner to mice in the negative control group.

### Mice sensitization and allergen-specific immunotherapy

Sensitization of mice was performed as described previously (11). Briefly, 20–22-week-old specific pathogen-free (SPF) male BALB/c mice (96 mice in total) were intraperitoneally injected twice at 14-day intervals with a 1000 PNU/200 µL concentration of *Artemisia vulgaris* (wormwood) pollen extract (Burli, Almaty, Kazakhstan) adsorbed on aluminum hydroxide (*In vivo*Gen; 1 mg/mouse). Mice from the negative control group (n=8) were similarly injected with PBS (200 µL). On day 21, all mice were challenged three times at one-day intervals (days 21, 23, 25) by intranasal administration of wormwood pollen extract (Burli) under ketamine-xylazine anesthesia at a dose of 200 PNU/20 μL or the same volume of PBS (negative control). Sensitization of mice to wormwood pollen was considered successful if at least 70% of the experimental animals had a significant increase of total and Art v 1-specific IgE compared with the negative control group.

**Figure 1:**
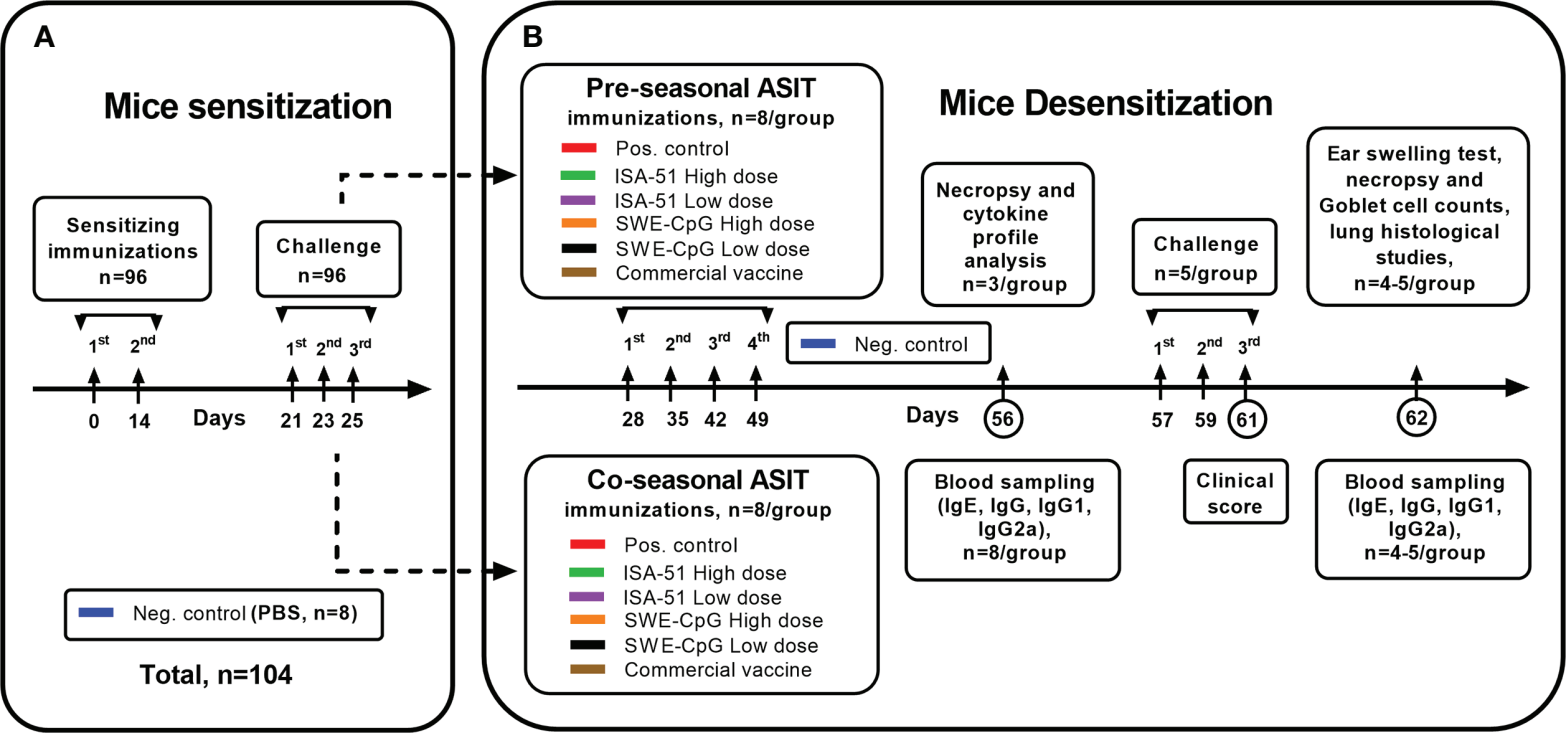
Study design. The schema of mice sensitization **(A)** by *Artemisia vulgaris* pollen extract and their desensitization **(B)** by vaccine formulations based on recombinant Art v 1 protein and various adjuvants using pre- and coseasonal ASIT regimens is shown.

On day 28, sensitized mice (**Figure 1**) were divided into two groups for pre- and co-seasonal ASIT, where animals were immunized 4 times at weekly intervals (days 28, 35, 42, 49) subcutaneously or intramuscularly with vaccine formulations in high and low dose regimens (**Table 1**, including commercial vaccine). Positive and negative control groups mice were intramuscularly injected with PBS in a similar manner. Co-seasonal ASIT mice underwent a single intranasal challenge with 200 PNU/20 μL of *Artemisia vulgaris* pollen extract (Burli) under ketamine-xylazine anesthesia for one hour after each vaccine immunization, as well as allergen nebulization. For allergen nebulization, mice (n=8) were transferred to a separate sealed chamber of 10 L volume and 1000 PNU of *Artemisia vulgaris* pollen extract (Burli) was sprayed there for 5 min using a FeelLife air pro 3 nebulizer (China). On day 56, serum (n=8/group) and spleen (n=3/group) samples were collected from all mice after pre- and co-seasonal ASIT to assess humoral (total and allergen-specific IgE antibodies, anti-Art v 1 IgG, IgG1, IgG2a antibodies) and cellular (Art v 1-specific cytokine profile) immune response factors.

To evaluate the effectiveness of ASIT, all mice (n=5/group) were subjected to triple allergy challenge (on days 57, 59, 61) by nebulization of *Artemisia vulgaris* pollen extract (1000 PNU/group, Burli) according to the previously described technique, and intranasal administration under ketamine-xylazine anesthesia of allergen in a dose of 200 PNU/20 µL. Mice from the negative control group (n=5) were similarly administered PBS instead of the allergen. During the third nebulization challenge (day 61), the mice were clinically monitored to assess the intensity of signs of allergic rhinitis (nasal rubbing and sneezing). After that, all mice had blood samples taken on day 62 for IgE, IgG, IgG1, IgG2a, an ear swelling test was done, and mice were then subjected to necropsy for histological analysis of the nasal turbinates and lungs.

### Determination of total IgE antibodies

Total IgE antibodies in µg/mL were determined using the ELISA MAX™ Standard Set Mouse IgE (Cat # 432401, BioLegend, USA) according to the manufacturer’s instructions.

### Determination of Art v 1- specific IgE antibodies

Allergen-specific IgE antibody determination was performed as previously described (11). Briefly, 96-well microplates were immobilized with 5 µg/10 mL (plate) of recombinant Art v 1 protein on an ELISA Coating Buffer (BioLegend) overnight. On the following day, ELISA Assay Diluent (BioLegend) in phosphate-buffered saline (PBS) was added into the plates at 200 μL/well and incubated on a PST-60HL thermal shaker (BIOSAN, Latvia) for 1 h at room temperature (RT). The plates were then washed four times with ELISA Wash Buffer (BioLegend). The mouse serum samples were diluted in a 1:5 ratio with ELISA Assay Diluent and 100 µl were added to the wells and incubated for 1.5-2 h at RT with stirring. After washing (4x), anti-mouse biotinylated detection antibodies for IgE (1:200, Cat # 406904, BioLegend, 100 µl/well) were added and the plates were incubated for 1 h at RT with stirring. After additional washing (4x), plates were incubated with HRP Streptavidin (BioLegend, 1:1000, 100 µL/well) for 30 min at RT with stirring. After that, the washing (5x) was repeated, and on completion of which the TMB substrate (BioLegend, 100 µl/well) was added. The color reaction was stopped by adding a stop solution (100 µL/well), and the optical density was measured at 450 nm on a Stat Fax 2100 analyzer (Awareness Tech).

### Determination of Art v 1-specific humoral immune response by ELISA

The ELISA procedure was performed in the same way as for the determination of Art v 1-specific IgE antibodies. For determination of IgG, IgG1, and IgG2a antibodies, mouse serum samples were serially two-fold diluted on ELISA Assay Diluent, starting from dilution 1:250 up to 1:8192000, and 100 µL were added to wells from each dilution and incubated under constant shaking for 1.5-2 h at RT. The secondary antibodies used were anti-mouse biotinylated detection antibodies for IgG (1:4000, Cat # 405303, BioLegend), IgG1 (1:1000, Cat # 406604, BioLegend), IgG2a (1:1000, Cat # 407104, BioLegend) in a volume of 100 μL/well. The HRP Streptavidin and TMB substrate were used in the same concentration as in the previous paragraph. The cut-off value for IgG, IgG1, and IgG2a antibody titers was calculated based on the average optical density (OD) of the wells containing only Assay Diluent (blank) + three standard deviations.

### Assessment of cellular immune response by cytokine profile

Cellular immunity was determined as previously described (11). Briefly, mice (n=3/group) were euthanized (cervical dislocation under ketamine/xylazine anesthesia) and their spleens were harvested under aseptic conditions. The spleen was mechanically crushed into single cell suspensions using a cell strainer (Falcon^®^ 70µm Cell Strainer). The grinding procedure was performed on a disposable sterile Petri dish (Piove di Sacco, Italy) with 10 ml of 3% fetal bovine serum (FBS, US Origin, Millipore Corp., Germany) in PBS. Erythrocytes were lysed with RBC lysis buffer (BioLegend). Splenocytes were cultured in a 5% CO_2_ incubator (INCO 153, Memmert, Germany) at 37°C in 24-well flat-bottomed plates (Sigma-Aldrich, USA) at a concentration of 1×10^6^ cells/well (1 mL) in RPMI-1640 + GlutaMax (Gibco) medium with 20 mM HEPES (Gibco), 10% FBS (inactivated by heating) and 1% Antibiotic-Antimycotic (Gibco™) in the presence of 10 μg of purified recombinant Art v 1 protein (AtaGenix) or without protein (control without stimulation). Cells were incubated for 48 h, after which the supernatant was examined for cytokines IL-2, IFN-γ, IL-13, TNF-α, IL-4, IL-5, IL-6, IL-9, and IL-10 using ELISA MAX™ Deluxe Set Mouse (BioLegend) kits, according to manufacturer instructions. Data were presented as the difference (delta) in cytokine concentration (in pg/mL) between samples with and without protein stimulation.

### Signs of allergic rhinitis and bronchial asthma in mice

Allergic rhinitis in *Artemisia vulgaris*-sensitized mice after nebulized challenge was assessed by clinical signs including sneezing and nasal rubbing (21). In histological analysis of mice nasal turbinates, the main sign of allergic rhinitis was the hyperplasia of Goblet cells in the transitional and respiratory epithelium (22). *Artemisia vulgaris* pollen-induced bronchial asthma in mice was evaluated by the ear swelling test and the level of pathological changes in the lungs by histological analysis.

### Counting of sneezing and nasal rubbing behaviors

After the third nebulization challenge of mice (n=5/group) with allergen, they were clinically observed for 10 min for signs such as nose rubbing and sneezing frequencies as described previously (21, 22). The experiment started with a negative control group, where PBS was sprayed in the chamber. After each allergen spraying, the chamber was washed and dried before being used for subsequent challenges. The mice were clinically observed by staff members who were blind to the study protocol.

### Allergy ear swelling test

For this test, 10 µl (100 PNU) of *Artemisia vulgaris* pollen extract was injected intradermally into the right auricle of the mice, with the negative control group injected in the same way with PBS. After 1.5-2 h, the thickness of both auricles was determined using an electronic digital micrometer MCC-25 DSWQ0-100II (China). The results of the ear swelling test were presented as the difference in thickness of the right (with allergen injection) and left (without injection) auricles, and expressed in mm.

### Histological analysis of mice nasal turbinates and lungs

Mouse nasal turbinates and lungs were fixed in 10% formaldehyde for 7 to 10 days after the excision process and washing in water, were subjected to histological wiring consisting of 4 portions of 100% isopropyl alcohol, and two portions of xylene. Nasal turbinates were decalcified in 20% EDTA-Na for 3 days. Then soaking of the material in 4 portions of paraffin and subsequent pouring of histological blocks were performed. After that, histological blocks were subjected to cutting on microprocessor-controlled microtome MZP-01 (KB Tekhnom, Russia), which resulted in 5 µm-thick sections. The sections were then deparaffinized in 2 portions of xylene and 3 portions of ethyl alcohol of decreasing concentration (96%, 80%, 70%), after which they were stained with hematoxylin (#05-002, BioVitrum, Russia) and eosin (#C0362, DiaPath, Italy). This was followed by clarification in ethyl alcohols of ascending strength (70%, 80%, 96%) and two portions of xylene. After completion, the sections were covered with coverslips using Bio Mount synthetic medium (#2813, Bio Optica, Italy). The preparations were studied using an Mshot microscope (China), model MF52-N. The photographs were made at x100 magnification using a Mshot MS23 camera head (China) in the MShot Image Analysis System (China). A x1000 magnification with an oil immersion lens was also used. A standardized scale was used for calibration. All measurements were made in μm. The number of mice nasal cavity Goblet cells was counted microscopically at x400 magnification in all sections of nasal turbinate mucosa (eosinophils were not detected). Pathological changes in the lungs of mice were evaluated according to a previously described point scale (11), the signs of which are grouped in **Supplementary Table**.

### Animal housing and bioethics

The entire cycle of work with laboratory animals was performed in the laboratory animals breeding vivarium (nursery) of the M. Aikimbayev National Scientific Center for Especially Dangerous Infections (NSCEDI) of the Ministry of Health of the Republic of Kazakhstan. Microisolator technology (microisolators) in individually ventilated complexes of Labproduct & Allentown (USA) was used as the basis for housing SPF animals. In accordance with national and international standards the laboratory animals were provided with access to feed and water *ad libitum* and optimal environmental conditions: air temperature 20-24°C, humidity 45-65%, illumination 325-350 Lx, noise level - no more than 60 dB, air volume per animal 0.25 m3/h, airflow rate 0.2 m/s, number of animals per cage - not more than 10, the minimum area of the cage - from 180 cm^2^, the norm of consumption of full-fed feed for adults - 12 g/head/day, for young - 5-8 g/head per day. Laboratory animals were provided with daily veterinary supervision, conditions to maintain a normal state of health, the ability to meet physiological and behavioral needs, and the rapid elimination of factors that could cause stress and suffering to the animals. To feed the animals we used full-fed autoclavable granulated feed for laboratory animals SSNIFF, standardized, enriched with vitamins, amino acids and minerals (62 elements), with at least 19-22% crude protein, without animal and growth supplements, without antibiotics, and with ISO 9001 quality certificate. Rehofix MK-2000 (JRS, Germany) autoclavable, dust-free, made of natural materials, with high absorption capacity, compatible with ventilated complexes, was used as bedding material. The animal study was reviewed and approved by the Institutional Animal Care and Use Committee (IACUC) of the NSCEDI according to Protocol #3 dated June 16, 2020. The IACUC operates under the laws of the Republic of Kazakhstan, the Directive 2010/63/EU on the protection of animals used for scientific purposes, and other internationally accepted standards which comply with the three Rs principle.

### Statistical analysis

GraphPad Prism 9.0.0 software (GraphPad Software, San Diego, CA, USA) was used for plotting and statistical analysis of the experimental data. Differences in antibody levels, cytokine production, ear swelling test results, clinical signs of allergic rhinitis, the number of Goblet cells, and pathological changes in the lungs between animal groups were evaluated using Tukey’s multiple comparisons test. The detection limit of IgG titers and its isotypes was 8.0 log_2_. For IgG, IgG1, and IgG2a antibodies, geometric mean titers with 95% confidence intervals were calculated and expressed in log_2_. We evaluated the relationship between the signs of allergic rhinitis and bronchial asthma in mice, as well as with various humoral and cellular immune response factors both after ASIT and challenge using the Pearson multivariable correlation method. For all comparisons, P<0.05 was considered a significant difference. All bars in the graphs represent the standard error mean.

## Results

### Reduction of levels of total and Art v 1-specific IgE

**Figure 2:**
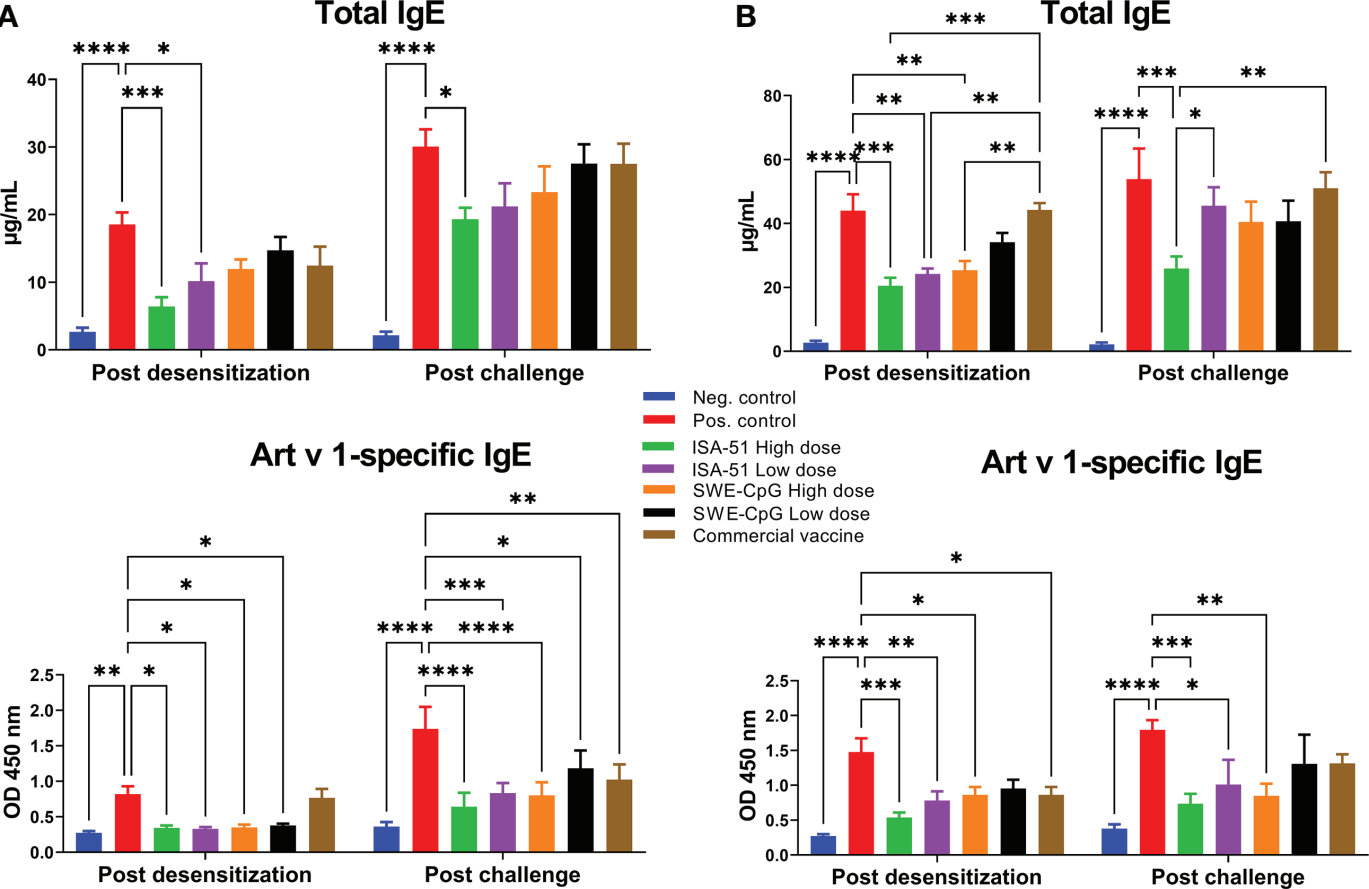
Reduction levels of total and Art v 1-specific IgE antibodies in *Artemisia vulgaris*-sensitized BALB/c mice after pre-seasonal **(A)** and co-seasonal **(B)** ASIT using different vaccine formulations. ASIT was performed with various vaccine formulations containing increasing high and low dose allergen (recombinant major *Artemisia vulgaris* pollen Art v 1 protein) and adjuvants such as ISA-51 and SWE-CpG. A commercial vaccine group was also included for comparison, comprising *Artemisia vulgaris* pollen extract formulated with aluminum hydroxide adjuvant. Challenge of mice after pre- and co-seasonal ASIT was performed by intranasal administration and nebulization of *Artemisia vulgaris* pollen extract. The concentration of total IgE antibodies is expressed as µg/mL. Art v 1-specific IgE antibody levels are shown as optical density values at 450 nm. Differences in IgE antibody levels between animal groups were assessed using Tukey’s multiple comparisons test. A P<0.05 value was considered a significant difference. *P <0.05, **P <0.01, ***P <0.001, and ****P <0.0001.

Only the recombinant Art v 1 protein-based vaccine formulated with ISA-51 adjuvant provided a significant reduction of total IgE levels in sensitized BALB/c mice compared to the corresponding positive control groups equally in high and low dose regimens of pre- and co-seasonal ASIT (**Figures 2A, B** **)**. With the SWE-CpG adjuvanted vaccine, significant reductions of total IgE were achieved only in the high-dose co-seasonal ASIT regimen (**Figure 2B**). The commercial vaccine did not significantly reduce total IgE, which were significantly higher than in the ISA-51 High dose, ISA-51 Low dose, and SWE-CpG High dose groups of mice after co-seasonal ASIT (**Figure 2B**). After challenge, significantly lower total IgE compared with the positive control group were only seen in the ISA-51 High dose group after both pre- and co-seasonal ASIT regimens (**Figures 2A, B** **)**. In the co-seasonal ASIT regimen after allergy challenge, mice in the ISA-51 High dose group also showed significantly lower total IgE compared with the ISA-51 Low dose and commercial vaccine groups (**Figure 2B**).

For Art v 1-specific IgE levels, almost all vaccine formulations tested including the commercial vaccine (but only after coseasonal ASIT), showed a significant decrease compared to the positive control groups after both pre- and co-seasonal ASIT regimens (**Figures 2A, B** **)**. Notably, significantly lower Art v 1-specific IgE (vs. positive group) after allergy challenge persisted in all experimental groups in the pre-seasonal ASIT regime (**Figure 2A**) as well as in the ISA-51 High dose, ISA-51 Low dose and SWE-CpG High dose groups after co-seasonal ASIT (**Figure 2B**).

In the co-seasonal ASIT regime, the levels of total and Art v 1-specific IgE in all groups (except for ISA-51 High dose group) were significantly higher compared to corresponding groups after pre-seasonal ASIT. Allergy challenge also contributed to a significant increase in total and Art v 1-specific IgE (except for ISA-51 High dose and SWE-CpG High dose groups) after pre-seasonal ASIT.

### Analysis of IgG antibody response after different ASIT regimens and challenge

**Figure 3:**
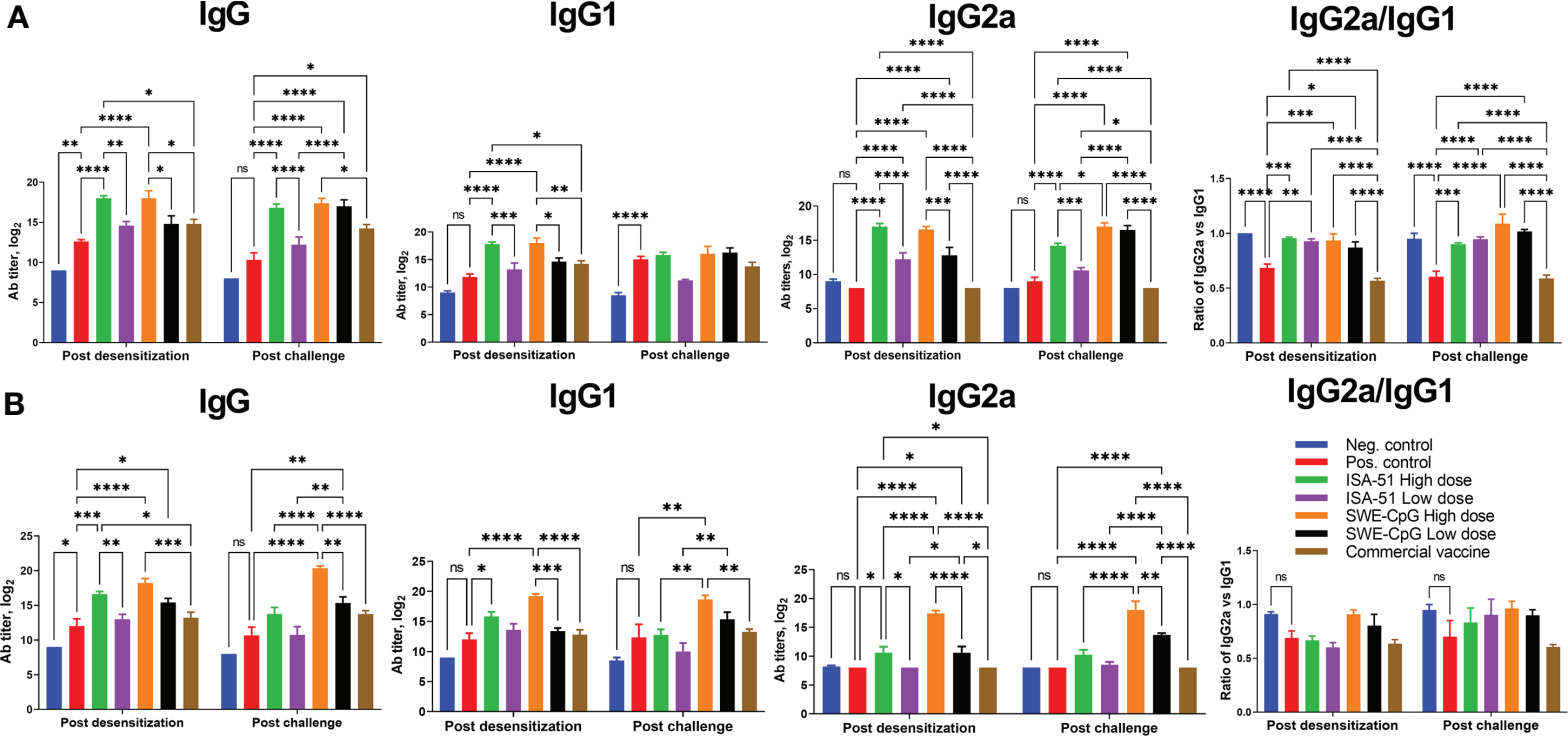
IgG, IgG1, and IgG2a ELISA titers as well as the ratio of IgG1 isotype antibodies to IgG2a in serum of BALB/c mice after pre- **(A)** and co-seasonal **(B)** ASIT and allergy challenge. ASIT was performed with various vaccine formulations containing high and low dose allergen (Art v 1 protein) and adjuvants (ISA-51 or SWE-CpG). A commercial vaccine group was included for comparison, comprising *Artemisia vulgaris* pollen extract formulated with aluminum hydroxide adjuvant. Challenge of mice after pre- and co-seasonal ASIT was performed by intranasal administration and nebulization of *Artemisia vulgaris* pollen extract. Antibody levels are presented as geometric mean titers with 95% confidence intervals and expressed in log_2_. Differences in antibody titers between animal groups were assessed using Tukey’s multiple comparisons test. A P<0.05 value was considered a significant difference. *P <0.05, **P <0.01, ***P <0.001, and ****P <0.0001.

Pre- or co-seasonal ASIT with all vaccine formulations (except the ISA-51 Low dose group for IgG and IgG1) induced significant Art v 1-specific IgG including its isotypes IgG1 and IgG2a (**Figures 3A, B** **)**. The high-dose vaccine formulations induced significantly higher IgG in most cases than the low-dose formulations. The commercial vaccine did not induce significant increases in IgG, IgG1, and IgG2a (vs. positive control) in the pre- and co-seasonal ASIT mice, with values significantly lower than the ISA-51 and SWE-CpG groups. Notably, the SWE-CpG adjuvanted vaccine formulations generated significantly higher IgG, IgG1, and IgG2a (particularly after allergy challenge) compared to the corresponding ISA-51 groups. Allergy challenge did not significantly increase or decrease IgG, IgG1, and IgG2a. For the ratio of IgG2a to IgG1, preferential polarization toward a Th1 response was observed in the ISA-51 and SWE-CpG groups with both dose regimens after pre-seasonal ASIT and allergy challenge, whereas Th2 responses predominated in the positive control and commercial vaccine groups (**Figure 3A**). In the co-seasonal ASIT and subsequent allergy challenge, all groups showed a similar, but less pronounced, pattern of IgG1/IgG2a ratios (**Figure 3B**).

### Analysis of cytokine profile after ASIT

**Figure 4:**
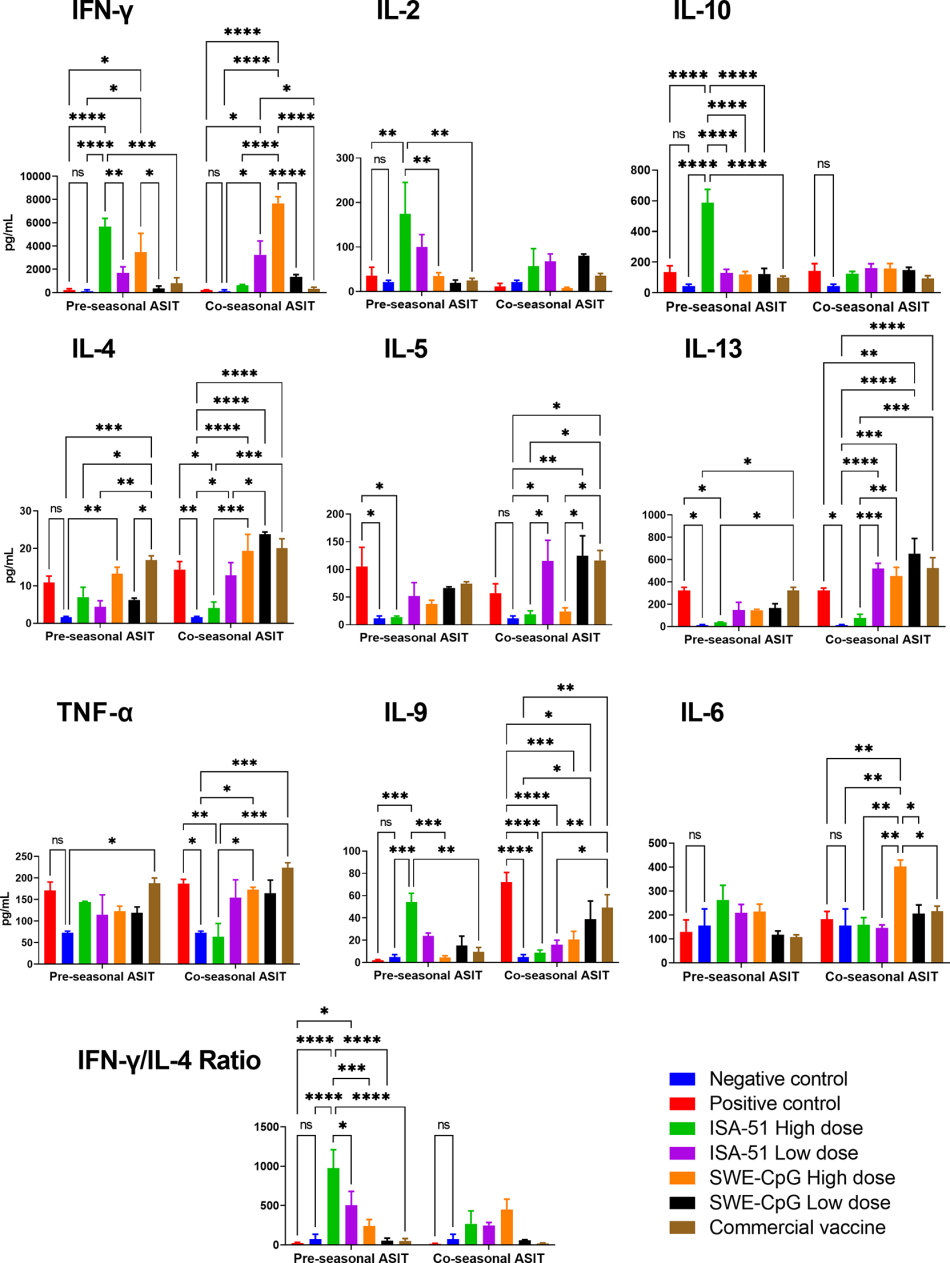
Art v 1 stimulated cytokine profiles in splenocytes of BALB/c mice after pre- and co-seasonal ASIT with different vaccine formulations. Cytokine data are presented as difference (delta) between samples with and without stimulation with Art v 1 protein and expressed in pg/mL. In addition, ratios between the IFN-γ and IL-4 are presented to assess Th1/Th2 balance. Differences between groups were assessed using Tukey’s multiple comparisons test. NS - not significant. A P<0.05 value was considered a significant difference. *P <0.05, **P <0.01, ***P <0.001, and ****P <0.0001.

In sensitized mice, depending on the ASIT regimen, the tested vaccine formulations elicited a highly diverse Art v 1-specific cytokine response in restimulated splenocyte suspensions (**Figure 4**). The ISA-51 adjuvanted vaccine formulations were the only ones that provided significant (vs. positive control) production of Th1-mediated cytokines IFN-γ and IL-2 in the pre-seasonal ASIT regimen, mainly at a high dose of antigen. The production of almost all Th2 cytokines (except IL-9) including IL-4, IL-5, and IL-13 was significantly lower in the ISA-51 High dose group than in the positive control group after pre-seasonal ASIT. In terms of the Th1/Th2 ratio as reflected by IFN-γ/IL-4, a Th1 polarized response was observed in the ISA-51 mice groups, especially in the high-dose pre-seasonal ASIT regimen. The ISA-51 High dose group also had the highest production of the Treg cytokine, IL-10, in the pre-seasonal ASIT regimen. In contrast, Th1 cytokines (IFN-γ; IL-2), were significantly lower after co-seasonal ASIT regime in the ISA-51 High dose group compared with those after pre-seasonal ASIT. The production of Th2 cytokines (IL-4, IL-5, and IL-13) in this group remained the same as after pre-seasonal ASIT. In the ISA-51 High dose group, the production of IL-9 and TNF-α was significantly lower not only in relation to the positive control, but also to the other experimental groups after co-seasonal ASIT regimen.

SWE-CpG-adjuvanted high-dose vaccine formulation in the pre-seasonal ASIT regimen induced significant production (vs. control) of both Th1 and Th2 cytokines (**Figure 4**). The SWE-CpG High dose (IFN-γ, IL-4, IL-13, IL-9, TNF-α, IL-6) and SWE-CpG Low dose (IL-4, IL-5, IL-13, IL-9) groups showed production of most cytokines in comparison not only to controls but also to those in the corresponding ISA-51 groups. The SWE-CpG High dose mice group after co-seasonal ASIT regimen showed significantly higher production of IL-6 than all other experimental groups. Overall, cytokine response levels in the SWE-CpG High dose (IFN-γ, IL-6, IL-13) and SWE-CpG Low dose (IL-4; IL-13) groups were significantly higher after co-seasonal ASIT than after pre-seasonal ASIT.

The positive control and commercial vaccine groups showed similar cytokine response profiles (**Figure 4**). In both groups of mice in the co-seasonal ASIT regimen, significant production of Th2 cytokines as IL-4, IL-5, IL-13, IL-9, as well as the proinflammatory cytokine TNF-α was observed compared to controls.

### Evaluation of allergic rhinitis therapy effectiveness in pre- and co-seasonal ASIT regimen with various vaccine formulations

**Figure 5:**
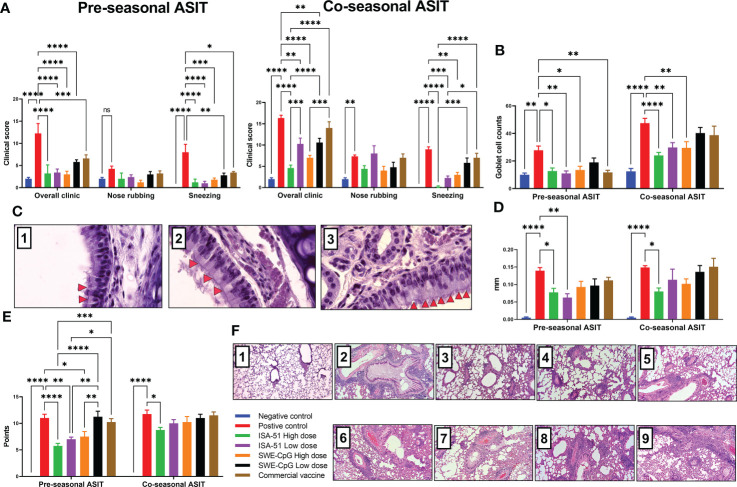
Therapy for *Artemisia vulgaris*-induced allergic rhinitis **(A–C)** and bronchial asthma **(D–F)** in pre- and co-seasonal ASIT regimen with different vaccine formulations. Allergic rhinitis in mice after challenge with *Artemisia vulgaris* pollen was evaluated by the intensity of such clinical signs as sneezing, nasal rubbing, and their combination **(A)**, and the number of Goblet cells in nasal mucosal epithelium by histological analysis **(B)**. **(C)** - histology of mouse nasal turbinates (Hematoxylin-Eosin staining; x1000): 1. Negative control, 2. ASIT group, 3. Positive controls. Red arrows indicate hyperplasia of the Goblet cells. The effectiveness of bronchial asthma therapy after ASIT was based on the results of the ear swelling test **(D)** and the level of lung pathological changes of mice **(E)** after challenge. The results of the ear swelling test are presented as the delta in the thickness of mouse auricles with the allergen or PBS, which was expressed in mm. Lung histological analysis after challenge was performed on a 16-point score (non-Type 2 inflammation). **(F)** - overview lung pictures of mice in different groups, x100 magnification: 1. Negative control; 2, 6. Positive controls in the pre- and co-seasonal ASIT regimens, respectively; 3, 7. ISA-51 groups in pre- and co-seasonal ASIT regimens, respectively; 4, 8. SWE-CpG groups in pre- and co-seasonal ASIT, respectively; 5, 9. Commercial vaccine group in pre- and co-seasonal ASIT regimens, respectively; Differences in the studied parameters between animal groups were assessed using Tukey’s multiple comparisons test. NS - not significant. A P<0.05 value was considered a significant difference. *P <0.05, **P <0.01, ***P <0.001, and ****P <0.0001.

All vaccine formulations, including the commercial vaccine, in the pre-seasonal ASIT regimen after challenge significantly reduced clinical signs (general and sneezing) of allergic rhinitis compared with the positive control group (**Figure 5A**). In the co-seasonal ASIT regimen, allergy challenge increased rhinitis clinical signs in all groups compared to those after pre-seasonal ASIT. However, despite this, in the ISA-51 High dose, ISA-51 Low dose, and SWE-CpG High dose groups, rhinitis clinical signs were significantly lower than in the positive control group. The lowest rhinitis clinical signs in the co-seasonal ASIT regime were observed in the ISA-51 High dose group mice. The clinical signs of rhinitis in the pre- and co-seasonal ASIT regimens correlated with the number of Goblet cells in the nasal mucosa (**Figures 5B, C** **)**. Thus, mice from the ISA-51 High dose, ISA-51 Low dose, SWE-CpG High dose, and commercial vaccine groups (pre-seasonal ASIT only) showed significantly lower Goblet cell hyperplasia on nasal mucosa compared to those of positive controls.

### Evaluation of bronchial asthma therapy effectiveness in pre- and co-seasonal ASIT regimen with various vaccine formulations

The efficacy of pre- and co-seasonal ASIT regimen in mice was evaluated after allergy challenge by an ear swelling test (**Figure 5D**), as well as by histological analysis of pathological changes in the lungs (**Figures 5E, F** **)**. Almost all groups in the pre-seasonal ASIT regimen showed a decrease in auricular swelling after allergen administration compared to the positive control group, but only in the ISA-51 High dose and ISA-51 Low dose groups was this difference significant. In the co-seasonal ASIT regimen, only the ISA-51 High dose group showed a significantly lower degree of ear swelling compared to the positive control (**Figure 5D**).

Lung histological analysis was performed to assess perivascular and peribronchial inflammation, as well as metaplasia of Goblet cells in the bronchi. The greatest lung pathological changes were observed in the positive control groups in both the pre- and co-seasonal ASIT regimens (**Figures 5E, F** **)**. A pronounced perivascular inflammation was observed, where large numbers of neutrophils and some eosinophils were present in the foci (data not shown). In addition, this group of mice showed moderate peribronchial inflammation with a small number of neutrophils and single eosinophils in the field of view. Metaplasia of Goblet cells was observed in most bronchioles (data not shown). Pulmonary pathological changes were also detected in all groups after challenge, but only in the ISA-51 High dose, ISA-51 Low dose, and SWE-CpG High dose groups the level of these changes were significantly lower in the pre-seasonal ASIT regimen than in the positive control, SWE-CpG Low dose, and commercial vaccine groups. In the co-seasonal ASIT regimen, only the ISA-51 high dose group showed significantly lower levels of lung inflammation compared with positive controls (**Figure 5E**).

### Correlation of effectiveness of allergic rhinitis and bronchial asthma therapy with different immune markers

**Figure 6:**
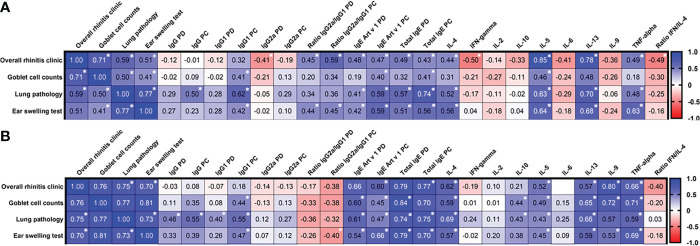
Correlation matrix analysis between allergic rhinitis and bronchial asthma signs and humoral and cellular immune variables after pre- **(A)** and co-seasonal **(B)** ASIT regimens and challenge. The color refers to the r value scale (from -1 to 1) shown on the right. The number in each cell represents the actual r value. The analysis was performed using Pearson’s multivariate correlation method. Normalized data were used for this analysis. PD – post desensitization, PC – post challenge. *P <0.05.

We evaluated the relationship of *Artemisia vulgaris*-induced allergic rhinitis (general clinical signs, number of Goblet cells on the nasal mucosa) and bronchial asthma (ear swelling test, lung pathology) with various humoral and cellular immune responses after pre- and co-seasonal ASIT regimens, and challenge (**Figure 6**). The results depended on the ASIT regimen. Thus, if in the pre-seasonal ASIT regimen there is a high degree of direct correlation within the specific to allergic rhinitis (clinic vs. Goblet cells, r=0.71, *P<0.0001*) and bronchial asthma (ear swelling test vs. pulmonary pathology, r=0.77, *P<0.0001*) signs, then in the co-seasonal ASIT regimen they all became highly correlated with each other (r=0.7-0.81, *P<0.0001*). Among humoral immune response factors in preseasonal ASIT regimen, only total IgE post challenge showed a high degree of correlation with the level of lung inflammation (r=0.74, *P=0.0013*). In the co-seasonal ASIT regimen, the correlation of total IgE with signs of allergic rhinitis (r=0.7-0.84, *P=0.015 - P<0.0001*) and bronchial asthma (r=0.7-0.79, *P=0.01 - P<0.0001*) became even more pronounced. These allergic signs also correlated with anti-Art v 1 IgE (r=0.6-0.66, *P=0.015 - P<0.0001*). Cytokine production levels in the pre-seasonal ASIT regimen had moderate to high correlation with allergic signs for IL-4 (r=0.52-0.56, *P=0.013-0.009*, vs. ear swelling test, lung pathology), IL-5 (r=0.63-0.85, *P=0.035-0.002*, vs. rhinitis, ear swelling test, lung pathology), IL-13 (r=0.68-0.78, *P=0.029-0.001*, vs. rhinitis, ear swelling test, or lung pathology), TNF-α (r=0.63, *P=0.002*, vs. ear swelling test), and highest negative correlation for IFN-γ and ratio of IFN-γ/IL-4 (r=-0.49-0.50, *P=0.022-0.024*, vs. rhinitis symptoms). In the co-seasonal ASIT regimen, only IL-9 (r=0.72-0.8, *P<0.0001*) and TNF-α (r=0.72-0.8, *P=0.001 - P<0.0001*) were significantly correlated with allergic rhinitis signs.

## Discussion

In earlier studies (11), we showed the efficacy against *Artemisia vulgaris* pollen-induced bronchial asthma of an ultrashort ASIT regimen with four immunizations at weekly intervals using recombinant Art v 1 protein formulated with ISA-51, Advax or SWE adjuvants. The ISA-51-adjuvanted formulation demonstrated the best efficacy and was selected for further development. Recombinant major *Artemisia vulgaris* pollen Art v 1 protein (expressed in *E. coli* bacterial cells), the natural form of which reacts with IgE of 95% of patients with wormwood allergy (23), was selected based on its hypoallergenicity (low binding to IgE) compared to pollen extract or natural protein, and its ability to induce a Th1 cellular immune response (24, 25). The oil adjuvant Montanide ISA-51 is a water-in-oil (W/O) emulsion of mineral oil and a surfactant from the mannide monooleate family (26). ISA-51 has previously been used in vaccine trials against such diseases as cancer, human immunodeficiency virus (HIV)/acquired immunodeficiency syndrome (AIDS) and malaria (27). A therapeutic lung cancer vaccine containing ISA-51 as an emulsion adjuvant with a quadruple immunization regimen at weekly intervals has been licensed in Cuba (28). ISA-51 has also been tested in influenza vaccine trials (29, 30).

For comparison, the present study included an ASIT vaccine formulated with the squalene-containing oil nanoemulsion adjuvant, SWE (Seppic, France) with the “oil in water” emulsion type, which in an earlier study (11) induced Art v 1-specific combined Th2/Th1 response and showed less therapeutic efficacy than the ISA-51-adjuvanted vaccine against bronchial asthma. In this study, we sought to enhance the Th1 polarization of the SWE-adjuvant by adding CpG55.2 oligonucleotide (Vaxine Pty Ltd, Australia) to the vaccine formulation, which stimulates Toll-like receptor 9 (TLR9) and activates humoral and cellular immunity (31). CpG55.2 in combination with Advax delta inulin adjuvant has already demonstrated its high efficacy in vaccines against tuberculosis (31, 32), Alzheimer’s disease (33) and Covid-19 (34).

This work is a continuation of research aimed to develop a vaccine for an ultrashort ASIT regimen for *Artemisia* pollen-induced allergic rhinitis and bronchial asthma. To achieve this goal, the previous vaccine based on Art v 1 protein with ISA-51 adjuvant was for the first time evaluated for simultaneous therapy of allergic rhinitis and bronchial asthma in *Artemisia vulgaris*-sensitized mice by ultrashort pre- and co-seasonal ASIT regimens using high and low-dose immunizations.

The tested vaccine formulations provided different therapeutic efficacy of allergic rhinitis and bronchial asthma in mice, based on the ASIT regimen and immunization dose. In the co-seasonal ASIT regime, *Artemisia vulgaris* pollen challenge induced more pronounced signs of allergic rhinitis, characterized by nasal rubbing and sneezing (21), hyperplasia of Goblet cells on nasal mucosa, and bronchial asthma (lung pathology with an ear swelling reaction) than in pre-seasonal ASIT. The pronounced allergic reactions in the co-seasonal ASIT regime was marked by higher levels of total and Art v 1 IgE (35), production of Th2-biased cytokines (IL-4, IL-5, IL-13, IL-9) (36–39) and TNF-α (40) (vs pre-seasonal ASIT). Most of the formulations tested had a therapeutic effect mainly during pre-seasonal ASIT, with only some improving co-seasonal ASIT. The effectiveness of therapy for allergic rhinitis was significantly greater than for bronchial asthma. *Artemisia vulgaris* pollen-induced bronchial asthma (11) was associated with lung pathological changes with predominantly neutrophilic non-Type 2 inflammation which is typical for rodents (41).

The ISA-51-adjuvanted vaccine formulation demonstrated the best efficacy in treating allergic reactions in mice with pre- and co-seasonal ASIT regimens. In the pre-seasonal ASIT regimen, this vaccine formulation provided a significant reduction in both allergic rhinitis and bronchial asthma signs (vs. positive control) in both the high- and low-dose regimens. The therapeutic effect of this ASIT regimen may reflect the observed pronounced polarization toward a Th1/Treg response. This was manifested by a significant decrease in the levels of total and Art v 1-specific IgE, but also by an increase in allergen-specific IgG2a/IgG1 and IFN-γ/IL-4, plus increased IL-10 production. In the co-seasonal ASIT regimen, similar results were achieved for allergic rhinitis, but only the ISA-51-adjuvanted vaccine in the high-dose group was successful against bronchial asthma. Interestingly, in this ISA-51 group, except for a significant decrease in IgE, no signs of a Th1 bias were observed. The therapeutic effect might instead reflect the observed reduction in Th2-immunity (IL-4, IL-9) and TNF-α.

The SWE-CpG-adjuvanted vaccine in high- and low-dose immunization schemes was also effective in the treatment of allergic rhinitis in the pre- and co-seasonal ASIT regimens. However, bronchial asthma therapy with this vaccine formulation was successful only in the pre-seasonal ASIT regimen with the high-dose regimen. The lower efficacy of this vaccine formulation compared to the ISA-51-adjuvanted vaccine may because despite the addition of the Th-1 polarizing CpG, as in the previous study there may still have been too much residual Th2 immunity (11).

The commercial vaccine in the ultrashort ASIT regimen showed the lowest effect in treating *Artemisia vulgaris* pollen-sensitized mice. While this vaccine had some effect on allergic rhinitis in the pre-seasonal ASIT regimen, and in the co-seasonal ASIT regimen, it was significantly less effective than the high dose ISA-51 and SWE-CpG vaccine formulations. The commercial vaccine group IL-4 and IL-13 responses were the same as the positive control group. Therefore, the treatment of allergic rhinitis with preseasonal ASIT with the commercial vaccine was associated with a decrease in Art v 1-specific IgE and an increase in blocking IgG. With respect to bronchial asthma therapy, the commercial vaccine was unsuccessful, and even though the natural treated *Artemisia vulgaris* pollen (allergoid) was used as an allergen. The reason for such low efficacy of the commercial vaccine may be explained by the presence of aluminum hydroxide adjuvant, which is effective only with a long-term ASIT regimen and with more than 4 injections (42).

The final stage of the study was to evaluate the correlation of allergic rhinitis and bronchial asthma with humoral and cellular immune parameters. This analysis showed a high degree of correlation of bronchial asthma with the severity of allergic rhinitis. The findings are in agreement with an earlier study (13), in which allergic rhinitis was an important risk factor for bronchial asthma. Among humoral immune response factors, total IgE was most strongly correlated with symptoms of allergic rhinitis and bronchial asthma. The data obtained are in agreement with our previous study (11). According to the cytokine profile in the preseasonal ASIT regimen, the development of allergic rhinitis was predominantly associated with IL-5 and IL-13 production, and bronchial asthma with IL-4, IL-5, IL-13, and TNF-α, which is consistent with other reports (36, 40). Interestingly, levels of IL-9 corelated with allergic reactions in the coseasonal ASIT regimen, which is also consistent with other reports (37–39). It is interesting that in preseasonal ASIT regimen, IL-9 production has a negative correlation with the manifestation of allergic reactions, which may be related to the induction of Th17 cells and in resolution of inflammation *via* Tregs (43). Notably in co-seasonal ASIT strongest negative correlation with allergy symptoms is with post challenge IgG2a/IgG1 which is stronger than IFN-γ/IL-4. This is different to pre-seasonal ASIT where IFN-γ has biggest effect and then IgG2a, which in mice according to (44) suppresses IgE-mediated allergic reaction.

Limitations of this study include limited group sizes and the fact the study has yet to be repeated. There is no way to know if the mouse model will replicate allergy to *Artemisia vulgaris* pollen in humans. In particular, the immune responses of mice differ significantly from human immune responses, and this may affect extrapolation of the study findings to humans. Nevertheless, this study highlights the importance of the recombinant allergen and adjuvant to the outcome of ASIT and highlights how they can be used to differentially modulate the immune response. Challenges remain around standardization of the potency and purity of allergens, and present a particular issue with natural extracts which can be highly variable (45). For this reasons, we elected to use a commercial recombinant protein as the allergen in these studies as this show allow for more consistency, with the antigen purity being greater than 90% and with a low endotoxin content that remaining within allowable regulatory limits for an injectable product. Besides purity, efficacy, potency and stability, safety and tolerability are key criteria for allergy vaccines. Although dose-dependent toxic side effects can be notable during allergen-specific immunotherapy, in our study even with high-dose ASIT vaccine formulations, we did not observe any toxicity or negative weight dynamics in the immunized animals. Preclinical safety studies (acute and chronic toxicity, allergenicity) of our ASIT vaccine in rats, mice and guinea pigs are currently being conducted at the testing laboratory of the National Center for Drug and Medical Device Expertise of the Ministry of Health (the Republic of Kazakhstan regulatory body). Evaluation of bronchial hyperreactivity was not performed in this murine model. An indirect method such as whole-body plethysmography or direct measurement (intratracheal) of airway hyperreactivity are necessary to support the immunotherapy scheme was effective in reducing asthma symptoms. This is a great limitation of the study.

We conclude that the recombinant major *Artemisia vulgaris* pollen Art v 1 protein-based vaccine formulated with the oil adjuvant ISA-51 in the ultrashort pre- and coseasonal ASIT regimens reduces signs of allergic rhinitis and bronchial asthma in sensitized mice. The findings provide support for further preclinical safety studies of the proposed vaccine candidate.

## Data availability statement

The original contributions presented in the study are included in the article/**Supplementary Material**. Further inquiries can be directed to the corresponding author.

## Ethics statement

The animal study was reviewed and approved by the Institutional Animal Care and Use Committee (IACUC) of the NSCEDI according to Protocol #3 dated June 16, 2020. The IACUC operates under the laws of the Republic of Kazakhstan, the Directive 2010/63/EU on the protection of animals used for scientific purposes, and other internationally accepted standards which comply with the three Rs principle.

## Author contributions

Conceptualization: KaisT, NP, TN; Data curation: KairT; Formal analysis: KaisT, NP, TN; Funding acquisition: KaisT; Investigation: KaiRT, MB, GF; Methodology: KaisT, MB; Project administration: KaisT; Resources: KaisT; Software: KaisT; Supervision: KaisT; Validation: KairT; Visualization: KaisT, NP, GR, MB; Writing ± original draft: KaisT; Writing ± review & editing: KaisT, NP, GR. All authors contributed to the article and approved the submitted version.

## Funding

This research was funded by the Science Committee of Ministry of Education and Science of the Republic of Kazakhstan (Grant No. AP08051924). Studies to evaluate vaccine formulations based on ISA-51 adjuvant were funded by the T&TvaX LLC. The funder was not involved in the study design, collection, analysis, interpretation of data, the writing of this article or the decision to submit it for publication. Development of CpG adjuvant was supported by funding from the National Institute of Allergy and Infectious Diseases of the National Institutes of Health under Contract HHSN272201400053C and HHSN272201800044C.

## Acknowledgments

The authors are grateful to Vaxine Pty Ltd (Australia) for kindly providing a sample of CpG55.2 adjuvant and to Seppic (France) for providing ISA-51 and SWE adjuvants. Special thanks to Turegeldiyeva D., Zhambyrbayeva L., Sarmantayeva K. for care and maintenance of laboratory animals. We also thank Temirzhan Nurpeisov and Saltabayeva U. for their valuable advice on the research findings.

## Conflict of interest

KaisT and KairT are affiliated with T&TvaX LLC. NP is affiliated with Vaxine Pty Ltd, a company holding rights to CpG adjuvant.

The remaining authors declare that the research was conducted in the absence of any commercial or financial relationships that could be construed as a potential conflict of interest.

## Publisher’s note

All claims expressed in this article are solely those of the authors and do not necessarily represent those of their affiliated organizations, or those of the publisher, the editors and the reviewers. Any product that may be evaluated in this article, or claim that may be made by its manufacturer, is not guaranteed or endorsed by the publisher.
