# Supplementary material for: A recombinant Artemisia vulgaris pollen adjuvanted Art v 1 protein-based vaccine treats allergic rhinitis and bronchial asthma using pre- and co-seasonal ultrashort immunotherapy regimens in sensitized mice
Source: Front Immunol. 2022 Nov 9;13:983621. doi: 10.3389/fimmu.2022.983621 (PMC9682083; doi:10.3389/fimmu.2022.983621)
Supplement: Supplementary file 1 [file Table_1.docx]

**Supplementary Table.** Scale for assessing pathological changes in the lungs of mice

| Evaluated trait | Points for the evaluated trait |
| --- | --- |
| Perivascular/peribronchial inflammation | 0 – no changes;  1 – moderate inflammation;  2 – pronounced inflammation;  3 – severe inflammation |
| Presence of neutrophils in foci of perivascular/  peribronchial inflammation | 0 – absent;  1 – less than 5 neutrophils per field with magnification (x1000);  2 – more than 5 neutrophils per field with magnification (x1000) |
| Presence of eosinophils in foci of perivascular/peribronchial inflammation | 0 – absent;  1 – single eosinophils on the field with magnification (x1000);  2 – multiple eosinophils on the field with magnification (x1000) |
| Metaplasia of the Goblet cells in the bronchi | 0 – absent;  1 – several Goblet cells are present in one or two bronchiolar profiles;  2 – numerous Goblet cells are present in bronchioles |
| Maximum score | **16** |
